# Factors Predicting Conviction in Stranger Rape Cases

**DOI:** 10.3389/fpsyg.2019.00526

**Published:** 2019-03-29

**Authors:** Samantha Lundrigan, Mandeep K. Dhami, Kelly Agudelo

**Affiliations:** ^1^ Anglia Ruskin University, Cambridge, United Kingdom; ^2^ Middlesex University, London, United Kingdom; ^3^ London Metropolitan Police Service, London, United Kingdom

**Keywords:** stranger, rape, jury decision-making, conviction, acquittal, real rape stereotype

## Abstract

**Background:** Despite there being no legal distinction between different types of rapes (e.g., those committed by strangers to the victim versus those committed by perpetrators known to the victim), stereotypical beliefs about rape have meant that these can be treated differently by the justice system. The aim is to explore the factors that predict juries’ decisions to convict or acquit in stranger rape cases.

**Methods**: We measured the importance of a range of 20 perpetrator-, victim-, and offense-related factors in predicting outcomes for 394 stranger rape cases tried by a jury. A four-stage analytic process was employed: (a) Kendall’s tau-b measured intercorrelations among the factors (predictors); (b) Chi-square and Welch *t*-tests measured associations between factors and verdicts; (c) binary logistic regression measured the power of factors in predicting verdicts; and (d) Stein’s formula was used to cross-validate the model.

**Results:** Jury verdicts were predicted by five offense-related factors and one victim-related factor. None of the perpetrator-related factors were significant predictors of convictions for stranger rape.

**Conclusion:** The findings have potential implications for victims of stranger rape, as well as prosecution and courtroom policy. We show that if a perpetrator is identified and charged, the likelihood of securing a conviction by a jury is high for victims of stranger rape. We suggest that prosecutors could gather as much information as possible from victims about the factors found to be of importance to juries, and judges could instruct juries on assumptions about the characteristics of the offense in order to challenge incorrect beliefs and stereotypes. Ultimately, this could be used to encourage victims of stranger rape to report and testify in court.

## Introduction

Few rape cases progress through the criminal justice system to trial (Beichner and Spohn, 2005; Kelly et al., 2005). For such cases, the prosecution must convince a jury that the perpetrator committed the crime. This can be challenging because rape cases often involve conflicting versions of events in the absence of strong corroborating evidence. In fact, jury conviction rates for rape are relatively low (Greenfeld, 1997; Temkin and Krahé, 2008; Munro and Kelly, 2009). For example, in England and Wales between 2006 and 2009, less than half (i.e., 47%) of offenders charged with rape of an adult female, who pleaded not guilty and were tried by jury, were subsequently convicted (Stern, 2010; Ministry of Justice, 2013). Furthermore, conviction rates for rape offenses have decreased, from 41% in 2012 to 36% in 2017 (Office for National Statistics, 2018). Convictions for rape are similarly low (e.g., 36%) in the USA (Bureau of Justice Statistics, 2013). The low conviction rates have especially negative ramifications for those victims who have defied low crime reporting rates for rape and endured the trial process, but failed to receive the justice they sought (Greenfeld, 1997; Stern, 2010).

There are, however, disparities in the conviction rates for different types of rape. For instance, scholars tend to agree that despite there being no legal distinction between rapes committed by strangers and rapes committed by acquaintances, these are often treated differently in the criminal justice system (Bryden and Lengnick, 1997). Rapes perpetrated by strangers are perceived as more serious, more likely to progress through the justice system, more likely to result in conviction, and more likely to receive harsher sentences than rapes perpetrated by someone known to the victim (Simon, 1996; Lovett and Kelly, 2009; see also Gottfredson and Gottfredson, 1988). Waterhouse et al.’s (2016) study of rape in a UK police force area found a conviction rate of 73% for stranger rape cases and 36% for acquaintance cases (see also, Grace et al., 1992; Gregory and Lees, 1996).

Efforts to understand how juries consider rape cases have often centered on the influence of rape myths, stereotypical beliefs that jurors might have about the victims and perpetrators of rape and the circumstances of such offences (e.g., Tetreault and Barnett, 1987; Tetreault, 1989; Eyssel and Bohner, 2010; McKimmie et al., 2014; See Dinos et al., 2015 for a review). In particular, researchers have proposed the existence of a “real” rape stereotype. It is suggested that in addition to a “stranger as perpetrator,” the elements of such a rape include a surprise approach, followed by a violent attack in an outdoor location, often with a weapon, with resulting injury to the victim (e.g., Estrich, 1987; Myhill and Allen, 2002; Temkin and Krahé, 2008; Munro and Kelly, 2009). Therefore, it is argued that rape cases which most closely correspond to this stereotype are more likely to result in conviction, whereas cases that deviate from the stereotype are less likely to be convicted (Willmott et al., 2018).

Surprisingly, there is a dearth of past research on stranger rape, despite it being a key feature of the “real” rape stereotype. Past studies attempting to identify the factors associated with conviction in rape cases have either only studied acquaintance rape (Fischer, 1995; Schuller and Wall, 1998; Wall and Schuller, 2000) or they have used the relationship between the victim and perpetrator as a variable in their analysis of a mixture of different types of rape (e.g., LaFree, 1980; Horney and Spohn, 1996; Munro and Kelly, 2009). These latter studies have demonstrated the importance of the stranger as perpetrator factor. However, analyzing stranger and non-stranger rapes together may inflate the importance attached to other elements of the “real” rape stereotype by virtue of their association with the “stranger as perpetrator” variable (e.g., an outdoor location may be more likely to be associated with stranger rather than non-stranger rape), rather than these elements being directly related to conviction. Furthermore, examining the two types of rape cases together may also introduce variability into the data that makes it difficult to identify factors associated with conviction for a specific type of rape. In support of this idea, McKimmie et al. (2014) examined the independent effects of offense type (stranger vs. acquaintance) and victim stereotypicality (resistance/no resistance vs. police cooperation/no cooperation) on mock jurors’ judgments and found that victim stereotypicality had greater influence on judgments in the acquaintance rape scenario than the stranger rape scenario. In the present study, we therefore examine jury convictions in stranger rape cases alone in order to better understand the factors that affect jury decision-making for such cases. Before presenting our study, we review relevant past research.

### Factors Associated With Conviction in Rape Cases

Theoretically, conviction in rape cases may be influenced by a myriad of factors, many of which can be subdivided into those related to the perpetrator, victim, or offense. Using either mock juror or criminal justice data, past research has examined how such factors are associated with, predictive of, or can explain case outcomes in rape cases. As noted above, past research does not typically examine stranger rape separately and we therefore review all research that has specifically examined case outcomes in (real or mock) rape cases.

#### Perpetrator-Related Factors

Beyond the perpetrator being a stranger that we have discussed above, several other perpetrator-related factors have been studied. These include age, ethnicity, previous convictions, and drug/alcohol intoxication at the time of offense. Two studies have reported a significant negative relationship between age and conviction (Spohn and Spears, 1996; Spohn and Horney, 2013) while two other studies have found no relationship (LaFree, 1980; Horney and Spohn, 1996). With regard to perpetrator ethnicity, one study has reported that black perpetrators are more likely to be convicted than white perpetrators (Feild and Bienen, 1980), another has shown the opposite (Spohn and Horney, 2013), and some studies have reported no relationship between the two factors (LaFree, 1980; LaFree et al., 1985; Horney and Spohn, 1996). Two studies found a significant positive relationship between the perpetrator’s previous convictions for any type of offense and conviction (Chandler and Torney, 1981; LaFree et al., 1985). Munro and Kelly (2009) found a positive relationship between the perpetrator’s previous convictions for sex offending and conviction. LaFree (1980) found a positive relationship between the seriousness of a perpetrator’s criminal history for sex offending (measured as no arrests, arrests but no convictions, or convictions) and conviction. Other studies, however, have found no relationship between the perpetrator’s criminal history and conviction (Horney and Spohn, 1996; Spohn and Spears, 1996; Spohn and Horney, 2013). Past studies have demonstrated a mixed and complex interplay between a perpetrator and victim’s level of intoxication and case outcome (Finch and Munro, 2004). For example, Wall and Schuller (2000) reported that when a victim was sober and a perpetrator was extremely intoxicated or when a victim and perpetrator were both moderately intoxicated, mock jurors were more likely to convict. By contrast, when a victim was portrayed as extremely intoxicated, perpetrator level of intoxication had no influence on conviction. However, other studies have found no relationship between perpetrator intoxication and case outcome (Fischer, 1995; Gunn and Linden, 1997).

#### Victim-Related Factors

Researchers have also studied a wide variety of victim-related factors. These can be grouped into demographic characteristics including age and ethnicity and factors describing the victim’s behavior around the time of the rape including drug/alcohol intoxication and the length of time taken to report to police. With regard to victim age, Grace et al. (1992) demonstrated that convictions were more likely in cases involving very young (<16 years) and older females (> 51 years). Kelly et al. (2005) showed that cases involving victims aged under 16 were more than twice as likely to result in guilty verdicts than cases where victims were aged 26–35 years. Campbell et al. (2009) found that cases involving victims between the ages of 18 and 21were significantly more likely to have their cases moved to depositions of higher outcomes (including conviction). However, the majority of studies have found no relationship between victim age and case outcome (LaFree, 1980; Horney and Spohn, 1996; Spohn and Spears, 1996; Gunn and Linden, 1997; Spohn and Horney, 2013). In relation to victim ethnicity, while two studies have reported that cases involving black victims are less likely to result in conviction (Feild, 1979; LaFree, 1980), the majority of studies have found no support for a relationship between victim ethnicity and case outcome (Feild and Bienen, 1980; LaFree et al., 1985; Horney and Spohn, 1996; Spohn and Horney, 2013). In relation to victim alcohol/drug intoxication, some studies have found a significant negative relationship between alcohol/drug intoxication and conviction (Wall and Schuller, 2000; Munro and Kelly, 2009), while others have found no relationship (Fischer, 1995; Gunn and Linden, 1997). The picture is further complicated when one considers the findings in relation to perpetrator intoxication (see above). Finally, past research has shown that the likelihood of conviction decreased as the time taken for the victim to report the crime increased (LaFree, 1980; Spohn and Spears, 1996; Spohn and Horney, 2013).

#### Offense-Related Factors

A number of offense-related factors have also been examined, including ethnic match between victim and perpetrator, number of perpetrators involved in the rape, location of attack, use of violence, and presence of weapon. Three studies have examined the relationship between perpetrator and victim ethnicity and case outcome. Feild (1979) found an interaction effect whereby black perpetrators were given longer prison sentences (used a proxy for verdict) by mock jurors but only if they were accused of attacking white victims. LaFree (1980) similarly found that black perpetrators were less likely to be convicted if a victim was black and more likely if a victim was white. By contrast, Spohn and Spears (1996) found that cases involving black perpetrators and white victims were less likely to result in conviction than cases involving white perpetrators and white victims. Of the two studies that have examined the relationship between the number of perpetrators and case outcome, LaFree (1980) found that cases involving more than one perpetrator were more likely to result in a guilty verdict than cases involving only one perpetrator. By contrast, Spohn and Spears (1996) found no relationship between the number of perpetrators and case outcome. Past research on the relationship between offense location and case outcome for rape cases has typically measured this variable as either indoors/outdoors or in a victim’s home or not. One study reported that a rape committed in a victim’s home was more likely to be convicted, although only if the perpetrator broke in (Kelly et al., 2005). Grace et al. (1992) found a higher conviction rate for offenses committed indoors than outdoors. However, other studies have found no evidence for an association between offense location and case outcome (LaFree, 1980; Horney and Spohn, 1996). Two studies have found a positive relationship between weapon use and likelihood of conviction (LaFree et al., 1985; Kelly et al., 2005) whereas one study found no such relationship (Spohn and Spears, 1996). A number of studies have demonstrated a significant positive relationship between the use of force or violence during an offense and conviction (e.g., Chandler and Torney, 1981; Grace et al., 1992; Du Mont and Myhr, 2000). By contrast, other studies have found no such relationship (Gunn and Linden, 1997; Spohn and Horney, 2013).

One of the reasons why some of the past research on the association between perpetrator-, victim-, and offense-related factors and outcomes in rape cases is mixed may be due to the fact that the data span several decades. Indeed, changes in the factors associated with the prosecution and/or conviction of rape cases were observed in Daly and Bouhours’ (2010) review of 33 studies that were published between 1970 and 2005. The studies were divided into two time periods (i.e., half published between 1970 and 1989 labeled “early” and half published between 1990 and 2005 labeled “later”). Together, the studies investigated eight broadly defined factors associated with prosecution or court decisions in rape cases. Two were perpetrator-related factors (i.e., stranger and criminal history), four were victim-related factors (i.e., age, character and credibility, injury/resistance, and promptness in reporting), and two were offense-related factors (i.e., forensic and witness evidence, and use of force/weapon). Daly and Bouhours coded the associations between the factors examined in each study and case outcome (i.e., prosecution and/or conviction). This resulted in 145 so-called “observations” representing a positive, negative, or no association between each factor and the outcome. In the early period, the factors most frequently significantly positively associated with conviction were a victim’s good character and credibility (broadly defined as no drugs/alcohol, no criminal convictions, and no risky behavior prior to rape; 82% of 11 observations); forensic or witness evidence (67% of 12 observations); victim injury/resistance (64% of 14 observations); and the stranger as perpetrator (54% of 13). The factors that were least frequently significantly positively associated with conviction were use of force/weapon (44% of 13 observations), suspect’s criminal history (43% of 7 observations), and a victim’s promptness in reporting (33% of 6 observations). However, this picture changed markedly in the later period, when the number of significant, positive associations between case outcome reduced for several factors, namely for the stranger as perpetrator factor (13% of 16 observations), victim’s good character and credibility (38% of 8 observations), and forensic/witness evidence (50% of 10 observations). By contrast, the number of significant, positive associations with conviction increased for other factors, namely victim injury/resistance (73% of 15 observations), suspect’s criminal history (67% of 3 observations), use of force/weapon (46% of 11 observations), and a victim’s promptness in reporting (38% of 8 observations)^1^.

#### Limitations of Past Research

In addition to the limitations outlined earlier, past research has some further shortcomings which may limit our understanding of why some stranger rape cases result in conviction, while others do not. Some past studies include both guilty pleas and guilty verdicts in their definition of conviction (e.g., Du Mont and Myhr, 2000; Kelly et al., 2005). These two types of decision (the decision by a perpetrator to plead guilty and the decision by a jury to convict) may be influenced by different factors. For example, past research has found that, beyond the predominant factor of strength of evidence, the decision to go to trial versus plead guilty can be influenced by a range of normative cognitive- and social-based pressures such as defendant overconfidence or denial, loss aversion, or social validation—factors that jurors are not necessarily subject to (Redlich et al., 2017). In addition, the evidence for some factors (e.g., perpetrator alcohol/drug intoxication) being predictive of, or associated with, case outcome in rape comes solely from studies of mock jurors. The external validity of these studies may be limited. Finally, as Daly and Bouhours (2010) have demonstrated, the relative importance of factors associated with conviction in rape cases can change over time. Updated research is pertinent when one considers the rape law reforms and policy and procedural reviews aimed at challenging views about rape that have occurred in recent years across several jurisdictions (Temkin and Krahé, 2008; Spohn and Horney, 2013). For example, in England and Wales over the last 20 years, there have been significant changes to rape law and policy. Such changes include the Sexual Offenses Act, 2003, one of the largest overhauls of sexual offenses in over a century, as well more recently a range of policy changes that have come about as a result of the CPS and Police National Rape Action Plan. In a recent study of UK specialist police officers’ decisions to progress rape cases, Dhami et al. (2018) found no evidence of officers’ reliance on factors such as the time taken to report the crime and victim’s alcohol/drug use during the offense, suggesting that recent changes in laws and policies may have altered police practice. It is unknown, however, to what extent such changes have filtered down to jury responses to rape.

#### The Present Study

The aim of the present study is to examine the power of a variety of perpetrator-, victim-, and offense-related factors in predicting jury convictions (defined as guilty verdicts) in stranger rape cases using recent data. Unfortunately, the lack of consistency in past findings and a lack of research on stranger rape alone preclude us from making *a priori* directional hypotheses about the relationship between specific perpetrator-, victim-, and offense-related factors and jury verdicts.

## Materials and Methods

### Ethics Statement

This study was conducted in accordance with the recommendations of Anglia Ruskin University Ethical Guidelines for research. The study was approved by the Humanities and Social Sciences Departmental Ethics Panel at Anglia Ruskin University. It is not considered necessary in research utilizing police data to seek the consent of those involved as the data are under the supervision of the police authority involved. All identifying information (e.g., names and addresses) was removed from records prior to the release of the data.

### Dataset

We analyzed data from the sexual offense database maintained by the Sexual Offenses Intelligence Unit of the UK London Metropolitan Police Service (LMPS). The database includes every sexual offense recorded within the LMPS area. The database contains information describing characteristics of the alleged perpetrator (where known), alleged victim, and the offense^2^.

The information in the database is obtained from case files that contain a number of documents (e.g., police reports, victim statements). The quality of information gathered from victims has benefitted from the introduction of dedicated police units specially trained in the investigation of rape complaints (Stern, 2010). In addition, in the present dataset, the average delay in reporting a crime was only 3 days. Specially trained analysts and researchers use an established coding dictionary when coding factors contained in the documents. This coding is also used in a number of other jurisdictions (e.g., USA, New Zealand) and all new analysts are required to undertake a rigorous data coding training program, utilize a “Quality Control Guide” to maximize consistency across analysts/researchers, and have their data inputting quality assured for the first 3 months in the unit.

### Sample

For present purposes, we extracted all of the adult^3^ (i.e., aged 16 or over), lone^4^ victim stranger rape cases, where the perpetrator and victim have no prior contact, reported to the LMPS between January 1st, 2001 and September 31st, 2015 where at least one defendant was tried by jury. The sample in the present study comprised 394 cases. Of these, 297 resulted in a conviction for rape^5^ (i.e., hereafter called rape-convicted) and 97 that resulted in an acquittal for rape (*n* = 97) (i.e., hereafter called rape-acquitted)^6^. The rape conviction rate in our sample was 75%.

### Factors

Based on the above review of the past literature and the availability of information contained in the database, 20 factors were included in the study^7^. These were grouped as follows: four perpetrator-related factors, four victim-related factors, and twelve offense-related factors (see Table 1).

**Table 1:** Factors and descriptive statistics.

Factor	% (N)	Mean (*SD*)	Range
**Perpetrator**			
**Age:** Perpetrator’s age at time of offense		27.05 (*8.02*)	14–53
**Ethnicity:** Ethnic group recorded as:			
*White*	37.83 (143)		
*Black*	48.15 (182)		
*Asian* *^1^*	14.01 (53)		
**Previous convictions** **^+^**			
*No convictions:* Perpetrator had no previous convictions	24.42 (73)		
*Previous convictions:* Perpetrator had any type of non-sexual convictions	58.86 (176)		
*Sexual convictions:* Perpetrator had previous sexual convictions	16.72 (50)		
**Alcohol/drugs**: Perpetrator had consumed alcohol/drugs prior to offense	7.11 (28)		
**Victim**			
**Age**: Victim’s age at time of offense		29.48 (*14.27*)	16–106
**Ethnicity:** Ethnic group identified with:			
*White*	79.90 (314)		
*Black*	12.21 (48)		
*Asian*	4.33 (17)		
*Chinese*	3.56 (14)		
**Alcohol/Drugs:** Victim had consumed alcohol/drugs prior to offense	38.07 (150)		
**Time taken to report:** Number of days taken to report offense		2.43 (*32.06*)	0–632
**Offense**			
**Age gap:** Perpetrator’s age in months less victim’s age in months		−1.58 (*15.76*)	−71 to 62
**Ethnic match:** victim and perpetrator same ethnicity	45.09 (170)		
**Approach style:**			
*Conversational:* Perpetrator spoke to victim prior to attack	40.10 (158)		
*Surprise:* No speech prior to attack	57.11 (225)		
*Blitz:* Sudden violence on attack	2.79 (11)		
**Number of perpetrators:** Number of perpetrators involved in offense		1.29 (*0.77*)	1–7
**Offense location:**			
*Outdoors*: Offense took place outdoors (i.e., a park or walkway)	51.52 (203)		
*Indoors:* Offense took place indoors (i.e., victim’s, perpetrator’s or other private dwelling, public building)	43.40 (171)		
*Vehicle*: Offense took place in a vehicle	4.31 (17)		
**Penetrative sexual contact:** Number of penetrative sexual acts committed (i.e. vaginal, oral, anal, digital and/or attempted penetration)		1.54 (*0.72*)	1–4
**Non-penetrative sexual contact:** Number of non-penetrative sexual acts committed (i.e., kissing, sexual touching, cunnilingus)		0.31 (*0.58*)	0–3
**Physical violence**: Perpetrator used any type of violence during the offense (e.g., hitting/punching, dragging, hair pulling, strangulation, gagging)	47.97 (189)		
**Verbal violence:** Perpetrator used verbal threats of violence toward victim	15.48 (61)		
**Weapon:** Perpetrator implied or used a weapon during offense	31.22 (123)		
**Theft of property**: Perpetrator stole from victim	27.41 (108)		
**Break-in:** Perpetrator broke into victim’s home	8.88 (35)		

1 Includes perpetrators of South East Asian descent including the countries of India, Pakistan, Bangladesh and Sri Lanka.

+ Missing data for 95 cases.

#### Perpetrator Factors

Four factors described a perpetrator’s demographic characteristics and behavior around the time of the offense. The demographic factors included age at time of the offense, ethnicity, and previous criminal convictions. The remaining factor described whether a perpetrator was thought to have consumed alcohol/drugs prior to the offense.

#### Victim Factors

Four factors described a victim’s demographic characteristics and behavior around the time of the offense. These were age at the time of offense (measured in months rather than years so as to provide a more refined measure of age), ethnicity, consumption of alcohol or drugs prior to the offense, and the number of days elapsed between the offense and reporting to the police.

#### Offense Factors

Twelve factors described the circumstances of the offense. One factor described the difference in age (measured in months) between a perpetrator and victim. Another factor described whether a perpetrator and victim were from the same ethnic group or not. The number of perpetrators involved in an offense was included as another factor. An approach style factor included three categories: conversational approach (perpetrator spoke to victim prior to attack e.g., “chatting up,” attempt to trick), surprise approach (no speech or physical violence on contact), or blitz approach (sudden violence on contact). An offense location factor had four categories: victim’s home, outdoors, perpetrator’s home, or public building. Two factors described the sexual behaviors committed by a perpetrator. A penetrative sexual contact factor recorded the total number (from 1 to 5) of five types of penetrative contact (i.e., vaginal penetration, oral penetration, anal penetration, attempted penetration, and digital penetration) that occurred during an offense. A non-penetrative sexual contact factor recorded the total number (from 0 to 3) of three types of non-penetrative sexual behaviors (i.e., kissing, sexual touching, and cunnilingus) that occurred during an offense. Two factors described the violent behaviors committed by a perpetrator. One described whether a perpetrator used any type of physical violence (e.g., hitting/punching, dragging, hair pulling, strangulation, gagging) during an offense and another factor described whether a perpetrator had used verbal violence (e.g., threats, obscene language) during an offense. One factor described whether a perpetrator had either used or implied a weapon of any kind (e.g., knife, blunt object) during the offense. One factor described whether a perpetrator stole personal belongings from a victim, and one factor described whether a perpetrator broke into a victim’s home.

## Analyses and Findings

Data analysis was conducted in four main steps. The first step involved measuring the associations among the perpetrator-, victim-, and offense-related factors. This was done using Kendall’s tau-b correlation, with a Bonferroni correction applied to the alpha level. The second step identified the perpetrator-, victim-, and offense-related factors associated with case outcome (i.e., conviction or acquittal). Here, we used the Chi-square test for dichotomous factors and the Welch *t*-test for factors measured on a continuous scale. Both types of analysis are suitable for use with unequal sample sizes. In the third step, we established the relative power of perpetrator-, victim-, and offense-related factors in predicting case outcome. Here, the factors found to be statistically significantly associated with case outcome that were identified in the preceding analysis were simultaneously entered into a logistic regression model. In the final step, we used Stein’s formula to cross-validate the model by calculating the adjusted *R* ^2^.

### Inter-Relations Among Perpetrator-, Victim-, and Offense-Related Factors

The size of the first-order intercorrelations among the factors ranged from −0.69 to 0.53 (*M* excluding sign = 0.19). Only 14 were statistically significant with a Bonferroni correction applied to the alpha level. The mean size of the statistically significant correlations was 0.32, excluding sign (*SD* = 0.15). The majority of the significant correlations were between factors that might be expected to be related to one another (e.g., age gap and age of victim/accused, ethnic match and victim/accused ethnicity; verbal violence and weapon, verbal violence and violence, weapon and theft). In addition, there were significant positive correlations between penetrative sexual acts and weapon (*r* = 0.20), victim age and break in (*r* = 0.22), and a significant negative relationship between weapon and victim alcohol/drugs (*r* = −0.27).

### Factors Associated With Conviction

#### Perpetrator Factors

Welch’s *t*-test was used to analyze one of the four perpetrator-related factors (i.e., perpetrator age), and Chi-square tests were used for the remaining three factors (i.e., perpetrator ethnicity, perpetrator alcohol/drug consumption, and previous convictions). The mean age of perpetrators in rape-convicted cases was 26.87 (*SD* = 7.98) and 27.62 (*SD* = 8.16) in rape-acquitted cases. This difference was non-significant, *t*(160) = 0.62, *p* = 0.60. The percentage of perpetrators belonging to the three different ethnic groups (i.e., white, black, Asian) was not significantly different between rape-convicted and rape-acquitted cases, χ*^2^* (1, *N* = 394) = 3.76, *p* = 0.15. The percentage of perpetrators who had consumed alcohol or drugs at the time of the offense did not differ significantly between rape-convicted cases (8.08%) and rape-acquitted cases (4.12%); χ*^2^* (1, *N* = 394) = 4.12, *p* = 0.26. The number of perpetrators with previous convictions also did not differ significantly between rape-convicted (non-sexual = 55.70%, sexual = 18.14%) and rape-acquitted cases (non-sexual = 70.97%, sexual = 11.29%); χ*^2^* (1, *N* = 299) = 4.75, *p* = 0.09.

#### Victim Factors

Of the four victim-related factors, two (i.e., victim age and the number of days before a victim reported to the police) were analyzed using Welch’s *t*-test, and two factors (i.e., victim ethnicity and drug/alcohol consumption) were analyzed using Chi-square tests. Two victim-related factors were found to be statistically significantly associated with case outcome. Specifically, victims in rape-convicted cases were older (*M* = 30.32, *SD* = 15.28) than victims in rape-acquitted cases (*M* = 26.88, *SD* = 10.22); *t*(245) = 6.35, *p* = 0.01. Rape-convicted cases (34.68%) were also significantly less likely than rape-acquitted cases (48.45%) to involve victims who had consumed drugs/alcohol at the time of the offense χ*^2^* (1, *N* = 394) = 5.88, *p* = 0.01, *d* = 0.12. Rape-convicted and rape-acquitted cases were not significantly different in relation to victim ethnicity, χ*^2^* (1, *N* = 394) = 2.77, *p* = 0.43, *d* = 0.08. The mean time a victim took to report the rape was 2.72 days (*SD* = 36.34) in rape-convicted cases and 1.56 days (*SD* = 6.00) in rape-acquitted cases. This difference was non-significant, *t*(340) = 0.27, *p* = 0.60.

#### Offense Factors

Four of the twelve offense-related factors (i.e., number of perpetrators, age gap between perpetrator and victim, penetrative sexual contact, and non-penetrative sexual contact) were analyzed using Welch’s *t*-test and the rest were analyzed using Chi-square tests. Six of the twelve offense-related factors were found to be statistically significantly associated with case outcome. Rape-convicted cases involved fewer numbers of perpetrators per case (*M* = 1.23, *SD* = 0.63) than rape-acquitted cases (*M* = 1.47, *SD* = 1.07); *t*(118) = 4.57, *p* = 0.03. Rape-convicted cases also involved greater numbers of penetrative sexual contact behaviors (*M* = 1.67, *SD* = 0.80) than rape-acquitted cases (*M* = 1.39, *SD* = 0.74); *t*(168) = 9.28, *p* = 0.003. Offense location was significantly associated with case outcome, χ*^2^* (1, *N* = 394) = 6.51, *p* = 0.03, *d* = 0.13. Examination of the adjusted standardized residuals for this factor revealed that an outdoor offense location was significantly more likely in rape-convicted cases (55.59%) than rape-acquitted cases (40.62%), (adjusted standardized residual = 2.5) and an indoor offense location was significantly less likely in rape-convicted cases (40.33%) than rape-acquitted cases (54.26%), (adjusted standardized residual = −2.4.). Of the two violence factors, rape-convicted cases were significantly more likely to involve verbal violence (19.19%) than rape-acquitted cases (4.12%); χ*^2^* (1, *N* = 394) = 12.68, *p* = 0.0001, *d* = 0.18. Rape-convicted cases were also significantly more likely to involve a used or implied weapon (34.68%) than rape-acquitted cases (20.62%); χ*^2^* (1, *N* = 394) = 6.73, *p* = 0.01, *d* = 0.13. Finally, theft of a victim’s property was significantly more likely in rape-convicted cases (33.33%) than rape-acquitted cases (9.28%); χ*^2^* (1, *N* = 394) = 21.26, *p* = 0.0001, *d* = 0.23.

The mean age gap between perpetrators and victims (i.e., perpetrator’s age minus victim’s age) in rape-convicted cases was −1.87 years (*SD* = 16.52) and −0.71 (*SD* = 13.21) in rape-acquitted cases. This difference was not significant, *t*(202) = 0.49, *p* = 0.48. There was no significant difference in the percentage of ethnically matched perpetrators in the rape-convicted cases (43.44%) and rape-acquitted cases (42.30%); χ*^2^* (1, *N* = 394) = 0.04, *p* = 0.91. There were also no significant differences in the style of approach between rape-convicted and rape-acquitted cases (conversational: 37.71 vs. 47.42%; surprise: 58.92 vs. 55.11%%; blitz: 3.37 vs. 10.03%); χ*^2^* (1, *N* = 394) = 3.85, *p* = 0.10. Lastly, there was no significance difference in the percentage of cases that involved a break-in between rape-convicted (9.43%) and rape-acquitted cases (7.22%); χ*^2^* (1, *N* = 394) = 0.44, *p* = 0.68.

### Factors Predicting Conviction

As revealed by the preceding analyses, a total of 8 of the 20 factors studied were significantly associated with case outcome. We used a logistic regression model to examine the utility of these factors in predicting case outcome. The eight factors (the offense location factor was recoded into two dummy variables: outdoor vs. indoor, outdoor vs. vehicle) were entered simultaneously into the model.

Table 2 presents the results of the regression analysis. A test of the full model against a constant only model was statistically significant, *χ^2^* (9) = 63.73, *р* < 0.001. Prediction success rose from 74.86 to 77.00%. This indicates that the set of predictors reliably distinguished between rape-convicted and rape-acquitted cases. A Nagelkerke’s *R* ^2^ of 0.24 indicated a moderate association between prediction and grouping. The Wald criterion demonstrated that six of the eight factors contributed significantly to the predictive utility of the model, from *p* = 0.002 to *p =* 0.044.

**Table 2:** Logistic regression model predicting stranger rape conviction.

Model	*B*	*SE*	Wald χ^2^	Odds ratio	95% CI		Lower	Upper
Victim age	0.02	0.01	4.43^*^	1.02	1.00	1.05
Victim alcohol/drugs	−0.47	0.28	2.89	0.62	0.36	1.08
Number perpetrators	−0.37	0.16	5.10^*^	0.69	0.50	1.00
Outdoors vs. indoors	−0.63	0.28	5.06^*^	0.54	0.31	0.92
Outdoor vs. vehicle	−0.12	0.65	0.03	0.89	0.25	3.18
Penetrative sexual behaviour	0.73	0.24	9.64^**^	2.08	1.31	3.30
Verbal violence	1.15	0.60	4.19^*^	3.14	1.05	9.40
Weapon	0.18	0.33	0.31	1.20	0.63	2.30
Theft from victim	1.27	0.40	10.40^**^	3.58	1.65	7.78
Constant	−0.06	0.55	0.01	0.94		

Note: ^*^ *p* < 0.05, ^**^ *p* < 0.01. Overall model: χ*^2^* (9, *N* = 394) = 63.73, *p* = <0.001, *R* ^2^ Nagelkerke = 0.24.

**Figure 1:**
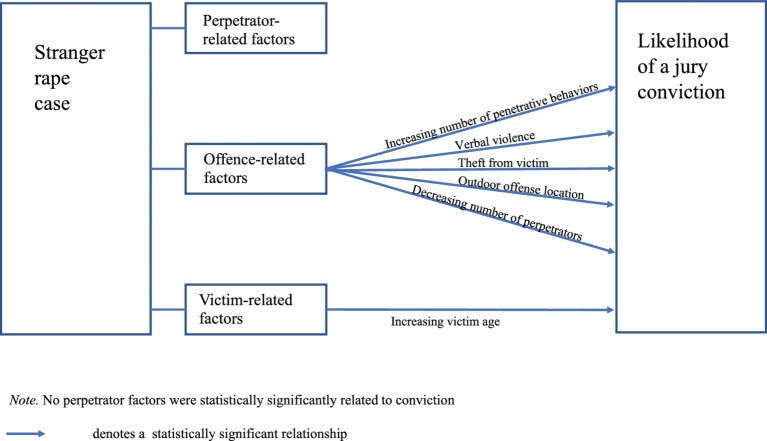
Predictive model of jury conviction in stranger rape cases.

Of these six factors, one was victim-related (i.e., age) and five were offense-related (i.e., number of perpetrators, offense location, verbal violence, penetrative sexual behavior, and theft from victim). Four factors increased the odds of conviction and the rest decreased the odds. Specifically, victim age increased the odds of conviction by 1.02 (or 2%, *p* = 0.035); for every additional penetrative sexual behavior, the odds of conviction increased by 2.08 (or 108%, *p* = 0.002); verbal violence increased the odds of conviction by 3.14 (or 214%, *p* = 0.015); and theft from the victim increased the odds of conviction by 3.58 (or 258%, *p* = 0.044). By contrast, increasing numbers of perpetrators in an offense reduced the odds of conviction by 0.69 times (or 31%, *p* = 0.024) and an indoor offense location reduced the odds by 0.54 times (or 46%, *p* = 0.025). Figure 1 summarizes the resulting predictive model of jury verdicts in stranger rape cases.

#### Cross-Validation of Model

The cross-validation of a model across different samples is an important test of its generalizability and consequently of its scientific value. There are two main cross-validation methods. The first, known as data splitting, involves randomly splitting a sample into a fitting and a validation sample. The regression model is developed using the fitting sample and then tested on the validation sample. An alternative approach, and the one employed here, is to calculate an adjusted *R* ^2^ that estimates the loss of predictive power (or shrinkage) were the model to be applied to a different dataset. One way to make this adjustment is to use Stein’s formula (see Equation (1)) where *R*^2^ is the unadjusted value, *n* is the sample size, and *k* is the number of predictors in the model (Field, 2009). Using this formula, we calculated an adjusted *R*^2^ of 0.22 for the regression model.

(1) $$\mathrm{Adjusted}\;R^{2}=1–\left[\left(\frac{n-1}{n-k-1}\right)\left(\frac{n-2}{n-k-2}\right)\left(\frac{n+1\;}{n}\right)\right]\left(1-R^{2}\right)$$

## Discussion

There is a dearth of research on how juries respond to stranger rape cases. The conviction rate of 75% observed in our dataset for such cases was substantially higher than the 36–47% reported in the past (Home Office, 2006; Stern, 2010). The latter figures refer to convictions in all types of rape cases, whereas the figure of 75% refers solely to stranger rape cases (and where there was no prior contact between the victim and perpetrator). This provides evidence to support the purported association between conviction for rape and the stranger as perpetrator factor (see also Simon, 1996; Bryden and Lengnick, 1997). This also further underscores the importance of studying different types of rape cases separately. Below, we summarize and discuss the findings of our study of jury responses to stranger rape.

### Predictors of Conviction in Stranger Rape

Eight of the factors examined in the present study were statistically significantly *associated* with case outcome (i.e., conviction or acquittal), and of these, six were statistically significant *predictors* of case outcome. As shown in Figure 1, our predictive model of jury conviction in stranger rape cases comprised one victim-related factor and five offense-related factors. Therefore, in the present study, none of the perpetrator-related factors were significant predictors of convictions for stranger rape. Our model suggests that although perpetrator-related factors may be of theoretical importance, and even shown to be important when examining either acquaintance rape or a mixture of different types of rape (e.g., Schuller and Wall, 1998; Wall and Schuller, 2000), in practice it appears that juries do not find perpetrator factors (i.e., those studied here) to be important in the context of stranger rape.

Of the victim-related factors studied, we found that just one of the four studied was a significant predictor in our model of stranger rape case outcomes. This is in contrast to a number of other studies (all of which have examined either acquaintance rape or a mixture of different types of rape) where conviction has been associated with a range of factors relating to perceived victim credibility, such as intoxication at the time of the offense (Wall and Schuller, 2000; Munro and Kelly, 2009) or the time taken to report to the police (LaFree, 1980; Spohn and Spears, 1996; Spohn and Horney, 2013). It has previously been argued that jurors may place less weight on victim-related factors and more on the circumstances of the rape and perpetrator-related factors in stranger rape cases (Ellison and Munro, 2010; McKimmie et al., 2014). Our findings lend support to that view.

We found that the likelihood of conviction increased with the age of the victim. There is some evidence to suggest that jurors perceive older victims to be more credible than their younger counterparts (Grace et al., 1992). Furthermore, victim age was significantly positively correlated with a perpetrator breaking into a victim’s home. It may be that juries are more convinced by cases involving a break-in to an older victim’s home. Our study focused solely on adult victims of stranger rape, whereas some past research examining the association between victim age and conviction in rape cases has combined both adult and child victims (i.e., 16 years or under), and studied them in the context of a mixture of different types of rape. This makes it difficult to compare the present findings with those of past research, particularly when past findings are themselves mixed. Campbell et al. (2009) found that conviction was more likely in cases with younger adult victims than older ones, whereas Horney and Spohn (1996) found no relationship between the age of victims and conviction. Studies including both child and adult victims have reported that either the odds of conviction are increased with reduced age of the victim (e.g., Kelly et al., 2005) or that there is no relationship between victim age and conviction (e.g., LaFree, 1980; Du Mont and Myhr, 2000; Stanko and Williams, 2009). Finally, Grace et al. (1992) reported that cases involving child victims or adults aged over 51 were more likely to result in conviction than cases involving victims between the ages of 16 and 51. Thus, further research separating adult and child victims and stranger from other types of rape is necessary.

We found that five offense-related factors were also significant predictors in our model of conviction in stranger rape case outcomes. An outdoor location was predictive of conviction. In fact, rape committed indoors had nearly a 50% *less* likelihood of being convicted than one committed outdoors. This finding is in contrast to previous studies that have either reported no relationship between offense location and case outcome (LaFree, 1980; Horney and Spohn, 1996; Du Mont and Myhr, 2000) or reported that offenses committed indoors, especially in a victim’s home are more likely to result in conviction (Grace et al., 1992; Kelly et al., 2005). Past research has combined stranger rape with other types of rape. One possible explanation for our finding may be the influence of the “real” rape stereotype (Estrich, 1987) where it is argued that rape cases which most closely correspond to this stereotype are more likely to result in conviction, whereas those that deviate from this stereotype are less likely to be convicted. Another possible explanation may be that offenses that occur outdoors could also be more likely to be witnessed thus adding evidential weight to the prosecution case.

In terms of how juries respond to the sexual behavior committed during a stranger rape, we found that increasing numbers of penetrative sexual behaviors were predictive of conviction. In fact, with each additional behavior, the odds of conviction increased by 108%. No previous research has examined the relationship between the number of penetrative acts involved in an offense and case outcome. One potential explanation for the present findings may be jurors’ expectations about the frequency with which particular offense behaviors occur in rape. Sleath and Woodhams (2014) reported that people significantly overestimated the frequency of a range of offense behaviors (including penetrative behaviors) that occur in stranger rape. It may therefore be that cases involving additional penetrative behaviors are less likely to violate jurors’ expectations about what a “real” rape looks like. Another explanation may be that the presence of more than one penetrative behavior indicates to jurors an increasingly serious and violent rape and so is more likely to result in conviction.

Unlike past research, we distinguished between both physical and verbal violence. We found that only verbal violence was predictive of conviction, and it increased the odds of conviction by over 200%. Verbal violence was positively correlated with both physical violence and weapon (used or implied), and this may explain the greater predictive power of verbal violence. The lack of predictive power of physical violence is worth noting because in previous studies, physical violence has been found to be positively associated with conviction in rape cases (e.g., Chandler and Torney, 1981; Grace et al., 1992; Du Mont and Myer, 2000). However, it would appear that the presence of physical violence alone is not enough to increase the likelihood of a conviction in stranger rape cases since we found that physical violence occurred in approximately half of convicted cases and half of acquitted cases. Nevertheless, we could not measure whether violence resulted in an injury and it may be that violence resulting in injury is associated with conviction.

We also found that theft of a victim’s property increased the odds of conviction by 258%. In our dataset, the victim’s property was stolen in just over a quarter of cases. However, despite its prevalence, no one to date has examined the association between theft of a victim’s property during a rape and case outcome. There are at least two possible explanations for why theft of a victim’s property may increase the likelihood of conviction in stranger rape cases. One is that theft is a criminal behavior, and so may be perceived by a jury as indicative of an individual’s general propensity for criminality. Another possible explanation is that the theft of identifiable property that is recovered may carry important evidential weight. Whereas the former factor is extra-legal and biases juries against the defendant, the latter is a legal factor that ought to be considered during trial.

Finally, we found that the greater the number of perpetrators involved in a stranger rape, the less likely a case was to be convicted, albeit by a small degree. This is contrary to an early study by LaFree (1980) who found that cases involving multiple perpetrators were more likely to result in a guilty verdict. One explanation for our finding is the diffusion of responsibility that may occur in multiple perpetrator cases which makes it more difficult for the prosecution to prove each defendant guilty beyond a reasonable doubt. Another related explanation is that it may be difficult for the prosecution to provide equal proof of guilt for all defendants in a case, thus making the whole case weaker.

#### Testing the “Real” Rape Stereotype

The “real” rape stereotype represents widely held and oversimplified beliefs about the conditions that are necessary for a rape to be perceived as “genuine” (Temkin and Krahé, 2008). Such beliefs can influence the decision-making of jurors (Temkin and Krahé, 2008) who, it is argued, are more likely to convict if the case resembles the “real” rape stereotype (Kelly et al., 2005; Hohl and Stanko, 2015). The empirical evidence to support this proposition, however, is limited. It relies on studies grouping together acquaintance and stranger rape cases, and explores a relatively small range of legal and extra-legal factors that might be associated with conviction. In addition, much of this research is now potentially outdated. We aimed to predict the outcomes of recent stranger rape cases tried by jury using a wide variety of victim-, perpetrator-, and offence-related factors.

The “stranger as perpetrator” factor comprises only one element of the “real” rape stereotype discussed in the extant literature (e.g., Estrich, 1987; Myhill and Allen, 2002; Temkin and Krahé, 2008; Munro and Kelly, 2009). The other elements are a surprise approach, a violent attack, an outdoor location, a weapon, and injury to the victim. We found that a surprise approach was not predictive of conviction in stranger rape cases. It may be that jurors recognize that stranger perpetrators can use a variety of tactics to approach their victims (Ellison and Munro, 2010). Weapon use was also not significantly associated with conviction. This is contrary to LaFree et al. (1985) and Kelly et al. (2005). However, both of these studies found that the association between weapon use and conviction held only under certain conditions (i.e., in the presence of injury or specific types of defense). Indeed, our finding is in line with other research suggesting that the presence of a weapon is not necessary for a jury to convict (e.g., Du Mont and Myhr, 2000; Campbell et al., 2009). As discussed above, an outdoor location and verbal, but not physical, violence were both predictive of case outcome. Therefore, the present findings only partially support the notion that the “real” rape stereotype influences jury decision-making in stranger rape trials.

### Strengths and Potential Limitations

The present study has several strengths. First, it is the first to focus exclusively on outcomes of actual *stranger* rape cases and so enables identification of some of the factors predictive of conviction and acquittal for this type of rape. Second, the present study focused on cases where a jury decided the outcome, whereas some past studies include both guilty pleas and guilty verdicts in their definition of conviction. It is important to examine these two routes to a conviction separately as they involve quite different decisions and decision-makers. Third, the present dataset spans a 15-year period up to 2015 and thus represents the most up-to-date analysis of conviction data since Lovett and Kelly (2009) who examined conviction data between 2001 and 2007. Social attitudes can change in response to social movements, legal policy reforms, or public awareness campaigns, thus making it important to update research findings and test the relevance of factors over time^8^. Fourth, the present study examined a variety of perpetrator-, victim-, and offense-related factors, including those not previously examined. Fifth, whereas previous research has typically measured either association or prediction between factors and case outcomes, we measured both. In addition, we reported intercorrelations among the factors, and cross-validated our regression model—analyses that have not been reported in the past studies reviewed above.

There are, nevertheless, some potential limitations of the present study. Most notably, the study focused only on factors available in the police database. Although we were able to explain 22% of the variation in outcomes for stranger rape cases, which is in line with previous studies (where reported) of between 15 and 30% (LaFree, 1980; LaFree et al., 1985), there are likely other factors predictive of case outcome that were not included in the present study due to the constraints of the data source. In particular, other factors found to be related to case outcome in rape include legal factors such as type and strength of evidence (e.g., Feild, 1979; LaFree, 1980; Campbell et al., 2009) and extra-legal factors such as juror attitudes (Lerner, 1980; Simonson and Subich, 1999; Schuller and Hastings, 2002). Future research ought to consider matching police datasets with court records to produce a more comprehensive dataset, although the analysis of some factors such as juror attitudes may still remain outside the scope of studies involving real case outcomes. Another potential limitation is that our data came from one urban geographic area of the UK. The representativeness of these findings to other, more rural areas ought to be explored in the future.

### Potential Implications for Policy and Practice

The present findings have potential implications for victims of stranger rape. We show that if a perpetrator is identified and charged, the likelihood of securing a conviction by a jury is high for victims of stranger rape. Furthermore, in arriving at a verdict, juries may focus less on the behavior and characteristics of the victim and more on the characteristics of the offense including the behavior of a perpetrator during the offense. This knowledge could be used to encourage victims of stranger rape to report crimes of rape and ultimately to testify in court.

There are also potential implications for how the police and prosecution services respond to stranger rape. First, these agencies could gather and present as much information as possible from victims about the factors found to be of importance to juries (i.e., offense-related factors), and pay less attention to those factors of lesser importance such as the victim’s behavior during the offense—which are also ones where recall may cause particular distress to victims. Second, the police and prosecution can manage their limited resources better when dealing with different types of rape cases. By contrast to acquaintance rape, stranger rape can be difficult to solve, but once a suspect has been identified, they are arguably more likely to result in a conviction. Thus, investigative resources could be better directed to solving stranger rape while prosecutorial efforts could more greatly emphasize case building for acquaintance rape. This might serve to reduce the disparity in outcomes for these two types of rape.

The present findings also have potential implications for courtroom policy. Since 2010, judges in England and Wales have been able to instruct juries on preconceived beliefs with the purpose of cautioning a jury against making unwarranted, pre-formed assumptions about the “behavior or demeanor of the complainant” (Section 13, p. 356, Crown Court Bench Book, 2010). This policy relates particularly to acquaintance rape but the present findings would suggest that in stranger rape cases, the focus might need to be elsewhere. Specifically, it may be necessary to instruct juries on assumptions about the characteristics of the offense—including the circumstances of the offense and the behavior of the perpetrator during the offense. In other words, it should not be assumed that stranger rape trials are immune to the effect of stereotypical, pre-conceived beliefs about what happens in a rape.

## Closing Remarks

Few rape cases progress through the criminal justice system to trial, and if they do reach the courtroom, jurors can be biased by stereotypical beliefs about “real” rape. This undermines the legal process, denies justice to victims, and potential rapists remain on the streets. The present study identifies elements of the “real” rape stereotype as it exists today when dealing with stranger rape cases. Future research ought to examine stereotypes around acquaintance rape and their role in conviction of these cases. Together, this body of evidence can serve to improve the effectiveness of the justice system’s response to all rape.

## Data Availability

The datasets for this study will not be made publicly available because it pertains to sensitive police recorded crime data.

## Author Contributions

SL contributed to research design, data analysis, interpretation, and intellectual content. MD contributed to research design, data analysis, interpretation, and intellectual content. KA contributed to acquisition of data, preparation of data, data analysis, and drafting of work.

### Conflict of Interest Statement

The authors declare that the research was conducted in the absence of any commercial or financial relationships that could be construed as a potential conflict of interest.

## Footnotes

^1^Victim age was not broken down sufficiently by either time period or type of criminal justice decision to allow for inclusion here.

^2^For ease of reading, hereafter, we use the terms ‘victim’ and ‘perpetrator’ even though some of the cases did not result in conviction.

^3^Cases involving child victims were excluded as past research suggests they may be influenced by different factors from cases involving adults.

^4^There were no cases of multiple victim rape in the dataset.

^5^Offenses of rape committed after 1 May 2004 in England and Wales are governed by the Sexual Offenses Act 2003. All references to the ‘Sexual Offenses Act’ in this section refer to this Act. The Sexual Offenses Act defines the offense of rape as follows: ‘Section 1

1. A person (A) commits an offense if—
  - he intentionally penetrates the vagina, anus or mouth of another person (B) with his penis,
  - B does not consent to the penetration, and
  - A does not reasonably believe that B consents
2. Whether a belief is reasonable is to be determined having regard to all the circumstances, including any steps A has taken to ascertain whether B consents.
3. Sections 75 and 76 apply to an offense under this section.
4. A person guilty of an offense under this section is liable, on conviction on indictment, to imprisonment for life.’

^6^An individual may be convicted of an offense other than rape where they have more than one charge against them in one offense (Criminal Procedure Rules, 2010).

^7^Factors were excluded on the basis of not being relevant to stranger rape or low frequency occurrence (<5% of cases).

^8^The data included offences defined both pre- and post- 2003 Sexual Offences Act. Statistical analysis showed no significant differences in findings for these two time periods.
